# Creatinine, Arsenic Metabolism, and Renal Function in an Arsenic-Exposed Population in Bangladesh

**DOI:** 10.1371/journal.pone.0113760

**Published:** 2014-12-01

**Authors:** Brandilyn A. Peters, Megan N. Hall, Xinhua Liu, Y. Dana Neugut, J. Richard Pilsner, Diane Levy, Vesna Ilievski, Vesna Slavkovich, Tariqul Islam, Pam Factor-Litvak, Joseph H. Graziano, Mary V. Gamble

**Affiliations:** 1 Department of Environmental Health Sciences, Mailman School of Public Health, Columbia University, New York, New York, United States of America; 2 Department of Epidemiology, Mailman School of Public Health, Columbia University, New York, New York, United States of America; 3 Department of Biostatistics, Mailman School of Public Health, Columbia University, New York, New York, United States of America; 4 Division of Environmental Health Sciences, School of Public Health and Health Sciences, University of Massachusetts, Amherst, Massachusetts, United States of America; 5 Columbia University Arsenic Project in Bangladesh, Dhaka, Bangladesh; National Centre for Scientific Research “Demokritos”, Greece

## Abstract

Kidney disease is emerging as an arsenic (As)-linked disease outcome, however further evidence of this association is warranted. Our first objective for this paper was to examine the potential renal toxicity of As exposure in Bangladesh. Our second objective relates to examining whether the previously reported positive association between urinary creatinine (uCrn) and As methylation may be explained by renal function. We had hypothesized that these associations relate to supply and demand for s-adenosylmethionine, the methyl donor for both creatine synthesis and As methylation. Alternatively, renal function could influence both As and creatinine excretion, or the As metabolites may influence renal function, which in turn influences uCrn. We conducted a cross-sectional study (N = 478) of adults, composed of a sample recruited in 2001 and a sample recruited in 2003. We assessed renal function using plasma cystatin C, and calculated the estimated glomerular filtration rate (eGFR). Consistent with renal toxicity of As, log-uAs had a marginal inverse association with eGFR in the 2003 sample (b = −5.6, p = 0.07), however this association was not significant in the 2001 sample (b = −1.9, p = 0.24). Adjustment for eGFR did not alter the associations between uCrn and the %uAs metabolites, indicating that GFR does not explain these associations. Increased eGFR was associated with increased odds of having %uInAs >12.2% (2001: OR = 1.01, 95%CI (1.00,1.03); 2003: OR = 1.04, 95%CI (1.01,1.07)). In the 2003 sample only, there was a negative association between eGFR and %uDMA (b = −0.08, p = 0.02). These results may indicate differential effects of renal function on excretion of InAs and DMA. Alternatively, a certain methylation pattern, involving decreased %InAs and increased %DMA, may reduce renal function. Given that these studies were cross-sectional, we cannot distinguish between these two possibilities. Discrepancies between the samples may be due to the higher As exposure, poorer nutrition, and lower As methylation capacity in the 2003 sample.

## Introduction

Approximately 140 million people worldwide are chronically exposed to inorganic arsenic (As) through contaminated drinking water, of whom approximately 57 million reside in Bangladesh [1]. In a survey of 4,997 tube wells conducted in 2000 in our study region of Araihazar, Bangladesh, 72% of wells exceeded the World Health Organization maximum contaminant level for As of 10 µg/L, and 52% exceeded the Bangladesh standard of 50 µg/L [2]. Exposure to inorganic As is associated with increased risk for cancers of the skin, lung, and bladder [3] as well as non-carcinogenic outcomes including cardiovascular disease, respiratory illness, and neurologic deficits [4]–[8].

Chronic kidney disease (CKD) is emerging as another As-induced disease outcome [9]. Elevated mortality rates from kidney diseases have been observed in ecological studies of As-exposed populations from Utah [10], Michigan [11], Taiwan [12], and Chile [13]. Cross-sectional studies have associated total urinary As (uAs) with increased odds of CKD [14], [15]. Animal studies suggest that As may cause kidney dysfunction through induction of oxidative stress in the kidney tissue [16]–[18]. Our first objective for this paper was to examine the potential renal toxicity of As exposure, measured by well water As and uAs (i.e. total uAs and uAs metabolites), in a cross-sectional sample of Bangladeshi adults. As a marker of renal function, we chose to measure plasma cystatin C. Very small reductions in glomerular filtration rate (GFR) increase serum cystatin C, making it a sensitive biomarker of GFR [19].

Our secondary objective for this paper relates to examining whether the previously reported associations between urinary creatinine (uCrn) and As metabolites may be explained by renal function. Arsenic metabolism, which facilitates uAs excretion, occurs through a series of methylation and reduction reactions which convert inorganic arsenic (InAs^V+III^) into monomethylarsonic acid (MMA^V^), monomethylarsonous acid (MMA^III^), and dimethylarsinic acids (DMA^V^) [20], [21]. The methylation reactions are catalyzed by arsenic methyltransferase (AS3MT) [22], [23], with S-adenosylmethionine (SAM) as the methyl donor. In urinary biomarker studies, uCrn concentration is widely used to adjust for urine dilution when measuring an analyte in a spot urine sample [24]. However, uCrn is not indicative only of hydration status, as it is also influenced by age, sex, race, BMI, and diet [24], [25], and in an As-exposed population in Bangladesh, we found that higher uCrn concentrations were associated with a significantly lower risk for As-induced skin lesions [26].

**Figure 1:**
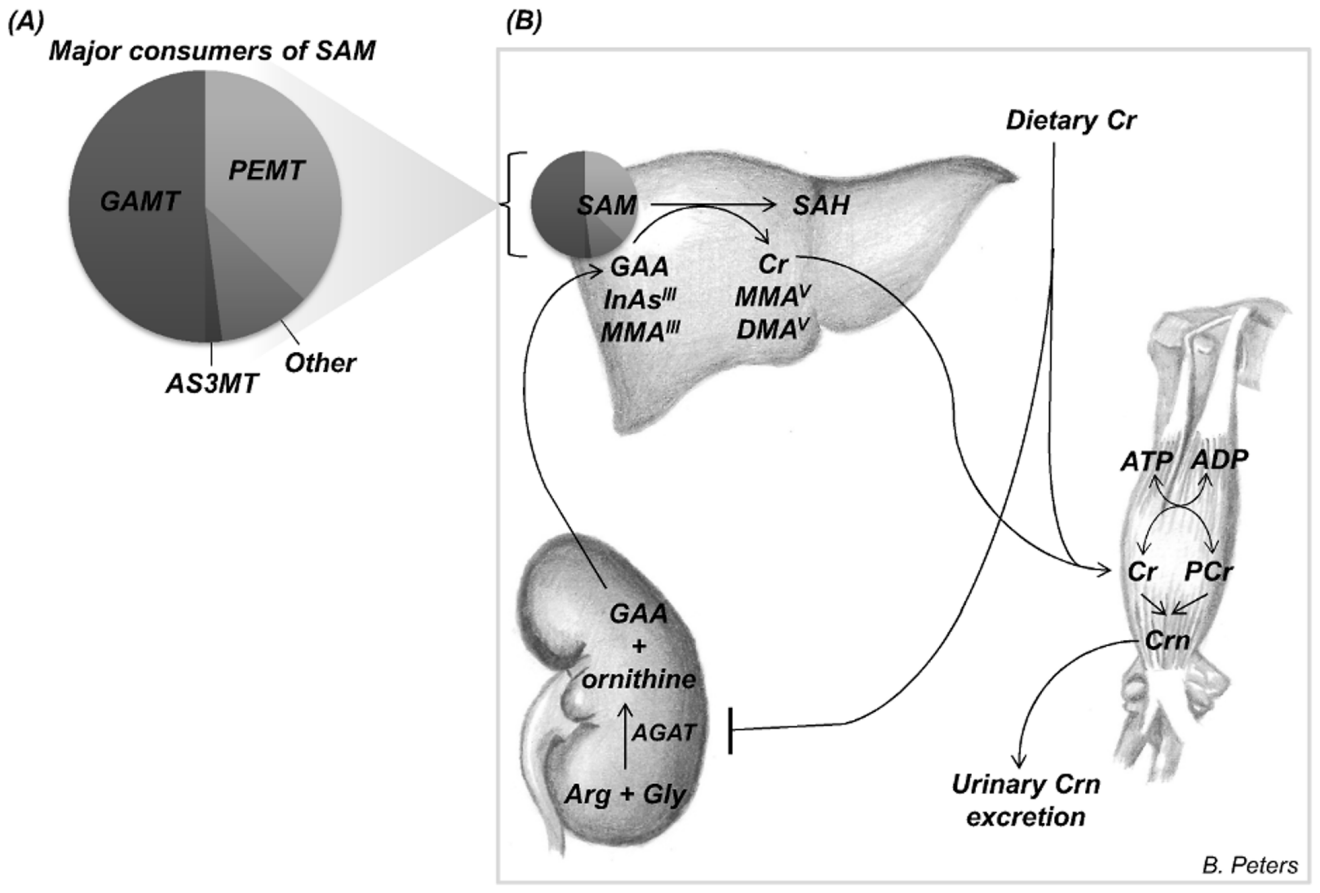
Arsenic metabolism and creatine synthesis. (A) Guanadinoacetate methyltransferase (GAMT) and phosphatidyl ethanolamine methyltransferase (PEMT), which catalyze the synthesis of creatine (Cr) and phosphatidylcholine, are the major consumers of S-adenosylmethionine (SAM). Arsenic methyltransferase (AS3MT) uses quantitatively much less SAM. (B) In the first, and rate-limiting, step of Cr biosynthesis, guanadinoacetate (GAA) is formed in the kidney by arginine:glycine amidinotransferase (AGAT). Dietary creatine (e.g. primarily from meat) leads to pre-translational inhibition of AGAT, thereby inhibiting endogenous creatine biosynthesis. GAA is transported to the liver, where it is methylated by GAMT to generate Cr, with SAM as the methyl donor. SAM also serves as the methyl donor for the methylation of trivalent inorganic arsenic (InAs^III^) to monomethylarsonic acid (MMA^V^), and for the methylation of monomethylaronous acid (MMA^III^) to dimethylarsinic acid (DMA^V^). The by-product of these methylation reactions is S-adenosylhomocysteine (SAH). Creatine, whether derived from endogenous biosynthesis or dietary sources, is transported to tissues with high energy requirements such as skeletal muscle, heart, and brain, where it is phosphorylated to phosphoryl-creatine (PCr). PCr is used for the regeneration of ATP during intensive exercise. Creatine and PCr are converted non-enzymatically at a constant rate to creatinine (Crn), which is then excreted in the urine. Image credit: Brandilyn A. Peters.

Our group [27]–[32] and others [33]–[35] have previously reported associations between uCrn and the percentage of total uAs that is inorganic As (%uInAs), and the percentage that is DMA (%uDMA). These studies have consistently shown that uCrn is positively associated with %uDMA, and negatively associated with %uInAs, however the mechanism underlying these observations is unclear. One explanation relates to the fact that creatine synthesis (**Figure 1**) consumes up to 50% of all SAM-derived methyl groups [36]–[38], while we estimate that methylation of a chronic high dose of As may consume only 2–4% [39]. In omnivores, roughly half of creatine requirements are met through dietary intake of creatine, primarily from meat [40]. Urinary creatinine is therefore a reflection of both dietary creatine intake and endogenous creatine synthesis [41], as well as hydration status. Studies in rodents have demonstrated that dietary creatine intake lowers creatine biosynthesis, thereby sparing methyl groups and lowering homocysteine (Hcys) [42]. We hypothesize that dietary creatine intake may also facilitate the methylation of As, and cause the observed associations between the %uAs metabolites and uCrn.

However, because uCrn levels may also be influenced by renal function [41], other potential explanations exist for the strong associations between the %uAs metabolites and uCrn. One is that renal function may differentially influence the excretion of InAs and DMA, thereby leading to a spurious association between uCrn and the %uAs metabolites (i.e. confounding). Alternatively, As metabolites may differentially influence renal function, which may then influence the excretion of creatinine (i.e. mediation). Remarkably, our understanding of the renal mechanisms of excretion of InAs is extremely limited [43]–[45] and, to the best of our knowledge, no published data are currently available regarding renal mechanisms of excretion and/or potential reabsorption of MMA or DMA. Additionally, the effect of individual As metabolites on renal function has not been thoroughly examined in the literature.

In summary, we had two objectives for this paper. First, we aimed to examine the potential renal toxicity of As exposure. Second, we aimed to examine the possibility that the associations between %uAs metabolites and uCrn may be explained by renal function. We carried out our objectives in a cross-sectional sample of Bangladeshi adults.

## Materials and Methods

### Ethics statement

This study was approved by the Institutional Review Board of the Columbia University Medical Center and the Bangladesh Medical Research Council. All participants gave written informed consent.

### Data availability statement

The data used for these analyses are available on request for academic, non-commercial purposes.

### Study participants

The Nutritional Influences on Arsenic Toxicity (NIAT) study [46] was a cross-sectional study of 1,650 adults randomly selected from the larger Health Effects of Arsenic Longitudinal Study (HEALS). HEALS is a prospective cohort study which originally recruited approximately 12,000 adults living in Araihazar, Bangladesh. The study region and recruitment of HEALS participants have been described previously [47]. Araihazar was chosen because it has a wide range of As concentrations in drinking water and is within a reasonable commuting distance from Dhaka.

### Study design

A subset of 300 people, representative of the larger cohort for total uAs, was selected from the NIAT study for measurement of uAs metabolites [30]. An additional random subset of 180 participants was later selected from the NIAT study for inclusion in the current study. Cystatin C was then measured in all N = 480 participants. Participants missing data on uAs metabolites (N = 2) were excluded from analysis, leaving us with a final sample size of N = 478. Recruitment for the NIAT study took place in two phases, in 2001 and in 2003, which corresponded with the baseline survey and first follow-up survey of the larger HEALS cohort, respectively.

### Collection of biospecimens

We obtained plasma samples by venipuncture after the participants had been sitting for 10–15 min. Blood was collected into EDTA-containing tubes and immediately placed in IsoRack cool packs (Brinkmann Instruments, Westbury, NY) designed to maintain samples at 0°C for >6 hr. Within 4 hr, samples were transported in hand-carried coolers containing additional ice packs to our local laboratory situated in our field clinic in Araihazar. Samples were centrifuged at 4°C and plasma separated from the cells. Plasma was then stored in aliquots at −80°C and shipped frozen on dry ice to Columbia University for analysis. Spot urine samples were collected into 50 ml acid-washed tubes, frozen at −20°C, and shipped on dry ice.

### Well water As

Field sample collection and laboratory analytical procedures are described elsewhere in detail [48], [49]. Water samples were collected in 20-mL polyethylene scintillation vials. The samples were acidified to 1% with high-purity Optima HCl (Fisher Scientific, Pittsburg, PA, USA) at least 48 hr before analysis [50]. Water samples were analyzed by graphite furnace atomic absorption (GFAA) with a detection limit of 5 µg/L. Samples found to have a concentration <5 µg/L were reanalyzed by high-resolution inductively coupled plasma mass spectrometry after 1∶10 dilution and addition of a Ge spike to correct fluctuations in instrument sensitivity. The detection limit of the method is typically <0.2 µg/L. A standard with an As concentration of 51 µg/L was run multiple times in each batch. The intra- and inter-assay coefficients of variation (CVs) for this standard were 6.01% and 3.76%, respectively.

### Total uAs and creatinine

Total uAs was measured by GFAA spectrometry using the AAnalyst 600 graphite furnace system (Perkin Elmer, Shelton, CT), according to the method of Nixon et al. [51]. The intra- and inter-assay CVs for total uAs were 2.5% and 4.7%, respectively. The laboratory participates in a quality control program run by the Institut de Sante Publique du Quebec, Canada. Intraclass correlation coefficients between our laboratory's values and samples calibrated at the Quebec laboratory were 0.99. We used a method based on the Jaffe reaction [52] to measure uCrn concentrations. The intra- and inter-assay CVs for uCrn were 1.9% and 4.9%, respectively.

### Urinary As metabolites

Arsenic metabolites were speciated using HPLC separation of arsenobetaine (AsB), arsenocholine (AsC), arsenate, arsenite, MMA^III+V^, and DMA^III+V^ followed by detection using ICP-MS [53]. The intra- and inter-assay CVs for InAs^III+V^ were 3.8% and 4.7%, for MMA^III+V^ were 4.6% and 8.9%, and for DMA^III+V^ were 2.0% and 1.9%, respectively. The percentages of InAs^III+V^, MMA^III+V^, and DMA^III+V^ were calculated using the sum of the inorganic and methylated metabolites as the denominator.

### Plasma cystatin C and eGFR

Cystatin C was measured by ELISA according to the manufacturer's protocol (R&D Systems Human Cystatin C Duoset Catalog# DY1196). We used a 6 point standard curve with a high standard of 3000 pg/ml. Samples were diluted 1∶2000 in PBS with 10% fetal bovine serum (Sigma Aldrich F6178). Recovery of the IFCC certified reference material for serum cystatin C (ERM-DA 471/IFCC) ranged from 104–114%. The intra- and inter- assay CVs were 3% and 10%, respectively. We calculated estimated glomerular filtration rate (eGFR) using the 2012 CKD-EPI Cystatin C equation [54].

### Plasma nutrients

Plasma folate and B12 were measured by radioimmunoassay (Quantaphase II; Bio-Rad Laboratories, Richmond, CA, USA) as previously described [46], [55]. The intra- and inter-assay CVs for folate were 3% and 11%, respectively, and those for B12 were 4% and 8%, respectively. Plasma total homocysteine (tHcys) concentrations were measured by high-performance liquid chromatography (HPLC) with fluorescence detection [56]. The intra- and inter- assay CVs for tHcy were 5% and 8%, respectively.

### Statistical analysis

Descriptive statistics were calculated for the total sample and by recruitment year, and differences between samples were detected using the Wilcoxon rank-sum test for continuous variables and the Chi-square test for categorical variables. We calculated prevalence of the 5 stages of CKD based on the National Kidney Foundation KDOQI guidelines as follows: Stage 1 is eGFR ≧90 ml/min/1.73 m^2^ + proteinuria, Stage 2 is eGFR 60–89 ml/min/1.73 m^2^ + proteinuria, Stage 3 is eGFR 30–59 ml/min/1.73 m^2^, Stage 4 is eGFR 15–29 ml/min/1.73 m^2^, and Stage 5 is eGFR <15 ml/min/1.73 m^2^. In examination of the bivariate associations between the %uAs metabolites and eGFR, we used partial Spearman correlation coefficients in order to eliminate the confounding effect of age and sex.

Regression models were used to examine the associations between a specific outcome variable and set of predictors, with and without control for potential confounding factors. Transformation was applied to variables with skewed distributions, to meet the assumptions of linear regression for the outcome variable, and to reduce the impact of extreme values in the predictors. Variables that underwent natural logarithmic transformation include %uInAs, age, uAs, and uCrn. The pattern of the associations between our predictors and outcomes of interest were examined by using linear models for continuous outcome variables and multinomial logistic regression analysis for categorized outcome variables.

For our first objective, to examine the potential renal toxicity of As exposure, we used linear regression models to examine the associations of either water As (wAs), total uAs, or speciated uAs metabolites, with the continuous outcome eGFR. We also examined logistic regression models using CKD (eGFR <60 ml/min/1.73 m^2^) as the outcome. *A priori* we decided to adjust for age and sex in these models, and uCrn in the models with total uAs or uAs metabolites as the predictors, to adjust for urine dilution. Other potential confounders (BMI, plasma folate, plasma B12, smoking, betel nut chewing, land ownership, education, systolic blood pressure, and diastolic blood pressure) were considered based on their bivariate associations with the main predictors (wAs, uAs, uAs metabolites), and the outcome (eGFR). The control variables in the final models were those that were associated with either of the main predictors and with the outcome (p<0.1), and resulted in an appreciable (>10%) change in the estimated regression coefficient for the association between a predictor and an outcome.

For our second objective, to determine whether the associations between the %uAs metabolites and uCrn are explained by GFR, we examined the correlations between eGFR and the %uAs metabolites, and the correlation between eGFR and uCrn. We then applied separate regression models using uCrn to predict each %uAs metabolite, with and without adding eGFR, and examined whether the parameter for uCrn was attenuated upon inclusion of eGFR. *A priori* we decided to adjust for age, sex, and uAs in these models. Selection of other potential covariates was done as described above.

Analyses were performed using SAS 9.1 (SAS Institute, Cary, NC). All statistical tests were two-sided with a significance level of 0.05.

## Results

Characteristics of the study participants for the total sample and stratified by recruitment year are presented in **Table 1**. The 2001 participants were slightly younger (p = 0.04), more educated (p = 0.09), had lower water As (p<0.0001), uAs (p = 0.005), and uAs per gram uCrn (p<0.0001), lower %DMA (p = 0.07) and lower eGFR (p = 0.02) as compared to the 2003 participants. The 2001 participants also appeared to be better nourished, with higher plasma folate (p<0.0001) and B12 (p = 0.0004) and lower plasma tHcys (p = 0.02). Since the 2001 sample was different from the 2003 sample on many factors, we present separate analyses for these groups in addition to the analyses for the total sample.

**Table 1:** Characteristics of the study samples.

	Total Sample (N = 478)		2001 Sample (N = 368)		2003 Sample (N = 110)		
	Mean ± SD	Range	Mean ± SD	Range	Mean ± SD	Range	P-value^a^
Age	36.4±10.1	18–64	35.9±10.1	18–64	38.1±10.2	18–64	0.04
Male (%)	43.93		44.84		40.91		0.47
BMI (kg/m^2^)^b^	20.2±3.2	13.9–33.3	20.2±3.2	13.9–33.3	20.1±3.1	14.6–28.4	0.65
Education (yrs)	3.8±3.9	0–15	4.0±3.9	0–14	3.3±3.7	0–15	0.09
Current smoker (%)	28.24		28.80		26.36		0.62
Betel Nut Use (ever) (%)	32.22		30.71		37.27		0.20
Water arsenic (µg/L)	96.5±104.3	0.1–648	86.1±104.8	0.1–648	131.5±95.2	0.5–504	<0.0001
Urinary arsenic (µg/L)	130.0±124.0	5–1026	125.0±125.3	5–1026	146.8±118.5	10–877	0.005
Urinary arsenic (µg/g Crn)	259.8±235.0	13.5–2363.6	244.8±242.4	13.5–2363.6	309.7±201.7	37.4–1013.7	<0.0001
Urinary creatinine (mg/dL)	63.2±49.3	4.4–311.0	64.3±48.8	4.4–311.0	59.3±51.0	6.4–274.8	0.19
%DMA^c^	71.9±9.0	17.8–96.5	71.5±9.1	17.8–96.5	73.0±8.6	41.9–87.5	0.07
%MMA^c^	12.9±5.2	0.6–28.8	13.0±5.2	0.58–28.8	12.5±5.1	3.1–26.8	0.23
%InAs^c^	15.2±7.4	0.1–79.1	15.5±7.8	0.05–79.1	14.5±5.5	5.2–32.0	0.25
Plasma folate (nmol/L)^d^	12.9±9.5	2.9–66.2	14.1±10.3	2.9–66.2	9.0±4.7	2.9–28.5	<0.0001
Plasma B12 (pmol/L)	286.6±112.9	79.1–1171.8	295.3±115.8	79.1–1171.8	257.3±97.6	97.0–676.5	0.0004
Plasma tHcys (µmol/L)^d^^,^ e	11.9±7.5	2.4–72.6	11.6±6.9	2.4–56.1	13.2±9.1	5.8–72.6	0.02
Plasma cystatin C (ng/mL)	982.9±317.9	438.0–4186.1	996.9±291.9	438.0–2393.1	935.9±390.5	471.8–4186.1	0.008
eGFR (ml/min/1.73 m^2^)	89.9±25.8	12.0–154.5	88.5±25.8	24.0–154.5	94.7±25.5	12.0–145.4	0.02
Proteinuria (%)^f^	6.90		7.07		6.36		0.80
Systolic blood pressure (mm Hg)^g^	112.2±15.7	60.0–183.0	111.9±15.2	60.0–168.0	113.2±17.3	79.0–183.0	0.76
Diastolic blood pressure (mm Hg)^g^	72.8±10.7	44.0–114.0	72.8±11.1	44.0–114.0	72.8±9.0	54.0–98.0	0.73
History of diabetes (%)^h^	2.20		2.62		0.91		0.29

a Chi-square and Wilcoxon's rank-sum tests were used to test for between sample differences in categorical and continuous variables, respectively.

b 2001 sample N = 366, 2003 sample N = 109.

c %DMA, %MMA, and %InAs, the proportion of total urinary arsenic excreted as dimethylarsinic acid, monomethylarsonic acid, and inorganic arsenic, respectively.

d 2003 sample N = 109.

e tHcys, total homocysteine.

f Defined as trace protein or greater in urine by dipstick test.

g 2001 sample N = 365.

h 2001 sample N = 344.

The prevalence of total CKD and individual stages of CKD by sample and gender are presented in **Table 2**. The prevalence of total CKD in the total sample was 19.87%; CKD prevalence was higher in the 2001 sample (21.20%) than in the 2003 sample (15.45%). Men in our study on average had lower eGFR, and a higher prevalence of CKD, than women (men: eGFR 80.12 ml/min/1.73 m^2^, CKD prevalence 28.10%; women: eGFR 97.62 ml/min/1.73 m^2^, CKD prevalence 13.43%), and this was true in both the 2001 and 2003 samples. Age, sex, smoking, and recruitment year were all independently associated with cystatin C in this population; these variables together accounted for 27% of the variation in cystatin C. As expected, cystatin C was also positively associated with plasma tHcys, likely due to the known influence of renal function on plasma tHcys concentrations [57].

**Table 2:** Prevalence of chronic kidney disease (CKD) stages by gender.

	Total Sample			2001 Sample			2003 Sample		
	Total (N = 478)	Men (N = 210)	Women (N = 268)	Total (N = 368)	Men (N = 165)	Women (N = 203)	Total (N = 110)	Men (N = 45)	Women (N = 65)
**Mean eGFR (ml/min/1.73 m^2^)**	89.93	80.12	97.62	88.51	78.90	96.31	94.70	84.58	101.71
**Total CKD (%)**	19.87	28.10	13.43	21.20	29.09	14.78	15.45	24.44	9.23
**Stage 1 (%)**	2.51	0.95	3.73	1.90	0	3.45	4.55	4.44	4.62
**Stage 2 (%)**	3.14	3.81	2.61	3.80	4.24	3.45	0.91	2.22	0
**Stage 3 (%)**	13.60	21.90	7.09	14.95	23.64	7.88	9.09	15.56	4.62
**Stage 4 (%)**	0.42	0.95	0	0.54	1.21	0	0	0	0
**Stage 5 (%)**	0.21	0.48	0	0	0	0	0.91	2.22	0

For our first objective, to assess the potential renal toxicity of As exposure, we examined linear regression models using water As, total uAs, or speciated uAs metabolites, to predict eGFR, adjusting for covariates (**Table 3**). Water As was not significantly associated with eGFR in the total sample, 2001 sample, or 2003 sample. In the total sample, the mean difference in eGFR for a one log-unit increase in total uAs was −2.55 ml/min/1.73 m^2^ (b = −2.55, p = 0.08); this effect size was between those of the two samples (2001 sample b = −1.90, p = 0.24; 2003 sample b = −5.62, p = 0.07). Log(uInAs in µg/L) was not associated with eGFR in the total sample, 2001 sample, or 2003 sample, while a one log-unit increase in uMMA was significantly associated with a mean decrease in eGFR of 2.55 ml/min/1.73 m^2^ (b = −2.55, p = 0.03) in the total sample. The negative association between log(uMMA) and eGFR was observed with marginal significance in both the 2001 sample (b = −2.13, p = 0.10) and the 2003 sample (b = −4.48, p = 0.06). Additionally, a one log-unit increase in uDMA was significantly associated with a mean decrease in eGFR of 6.42 ml/min/1.73 m^2^ in the 2003 sample (b = −6.42, p = 0.04), although this association was not significant in the total sample or 2001 sample. These results were similar without adjustment for urinary creatinine (data not shown). In our logistic regression analysis with CKD (eGFR <60 ml/min/1.73 m^2^) as the outcome, we did not observe that total uAs, uAs metabolites, or water As were associated with increased odds of CKD (data not shown).

**Table 3:** Linear regression models using log(total urinary As in µg/L), log(uAs metabolites in µg/L), or log(water As in µg/L) to predict eGFR.

	Total Sample (N = 478)			2001 Sample (N = 368)			2003 Sample (N = 110)		
Predictor	B (SE) ml/min/1.73 m^2^	p-value	R^2^ (%)	B (SE) ml/min/1.73 m^2^	p-value	R^2^ (%)	B (SE) ml/min/1.73 m^2^	p-value	R^2^ (%)
**Log(Urinary As)** a	−2.55 (1.44)	0.08	29.51	−1.90 (1.62)	0.24	26.57	−5.62 (3.04)	0.07	41.95
**Log(uInAs)** a	−0.66 (0.98)	0.50	29.11	−0.77 (1.07)	0.47	26.40	0.21 (2.52)	0.93	40.05
**Log(uMMA)** a	−2.55 (1.14)	0.03	29.79	−2.13 (1.30)	0.10	26.83	−4.48 (2.35)	0.06	42.08
**Log(uDMA)** a	−2.24 (1.40)	0.11	29.42	−1.39 (1.58)	0.38	26.45	−6.42 (3.03)	0.04	42.54
**Log(Water As)** b	−0.67 (0.56)	0.23	29.22	−0.59 (0.60)	0.32	26.44	−1.44 (1.77)	0.42	40.35

a Adjusted for log(age), sex, current smoking, log(urinary creatinine), and recruitment year (total sample only).

b Adjusted for log(age), sex, current smoking, and recruitment year (total sample only).

For our second objective, we aimed to determine whether the associations between uCrn and %uAs metabolites may be explained by GFR. Consistent with previous findings, in the total sample the partial Spearman correlations controlling for age, sex, and recruitment year indicated that uCrn was inversely correlated with %uInAs (r = −0.38, p<0.0001) and %uMMA (r = −0.15, p = 0.0009), while positively correlated with %uDMA (r = 0.35, p<0.0001) (**Table 4**). These partial correlations were consistent between the 2001 and 2003 samples.

**Table 4:** Partial Spearman correlation coefficients for associations between % urinary As metabolites and urinary creatinine, cystatin C, and eGFR, adjusting for sex and age (and recruitment year in the total sample only).

	%InAs	%MMA	%DMA
**Total Sample (N = 478)**			
Urinary creatinine (mg/dL)	−0.38 (<0.0001)^a^	−0.15 (0.0009)	0.35 (<0.0001)
Plasma cystatin C (ng/ml)	−0.13 (0.005)	0.04 (0.43)	0.05 (0.28)
eGFR (ml/min/1.73 m^2^)	0.12 (0.007)	−0.03 (0.50)	−0.05 (0.29)
**2001 Sample (N = 368)**			
Urinary creatinine (mg/dL)	−0.34 (<0.0001)	−0.13 (0.02)	0.32 (<0.0001)
Plasma cystatin C (ng/ml)	−0.10 (0.05)	0.03 (0.53)	0.04 (0.47)
eGFR (ml/min/1.73 m^2^)	0.10 (0.06)	−0.03 (0.62)	−0.04 (0.45)
**2003 Sample (N = 110)**			
Urinary creatinine (mg/dL)	−0.51 (<0.0001)	−0.24 (0.01)	0.43 (<0.0001)
Plasma cystatin C (ng/ml)	−0.21 (0.03)	0.03 (0.75)	0.09 (0.33)
eGFR (ml/min/1.73 m^2^)	0.20 (0.04)	−0.04 (0.68)	−0.08 (0.43)

a r (p-value), all such values.

Plasma cystatin C had a negative partial correlation with %uInAs (r = −0.13, p = 0.005), and this was consistent between the 2001 and 2003 samples (**Table 4**). Likewise, eGFR had a positive partial correlation with %uInAs (r = 0.12, p = 0.007). Urinary %MMA and %uDMA were not associated with plasma cystatin C or eGFR. eGFR was not associated with uCrn in the total sample (Spearman r = −0.05, p = 0.30), 2001 sample (Spearman r = −0.07, p = 0.21), or 2003 sample (Spearman r = 0.01, p = 0.92). This result did not support the hypothesis that GFR may serve as a confounder or a mediator of the associations between uCrn and the %uAs metabolites. Nevertheless, we did examine regression models using uCrn to predict each %uAs metabolite, adjusting for total uAs, age, sex, smoking, and recruitment year, with and without adjustment for eGFR (**Table 5**).

**Table 5:** Logistic and linear regression models using uCrn and eGFR to predict %uAs metabolites.

			Total Sample (N = 478)			2001 Sample (N = 368)			2003 Sample (N = 110)		
**Logistic models**											
**Outcome**	**Model** a	**Predictor**	**OR (95% CI)**	**P**	**R^2^ (%)** b	**OR (95% CI)**	**P**	**R^2^ (%)** b	**OR (95% CI)**	**P**	**R^2^ (%)** b
**%uInAs** c	Model 1	Log (Urinary Creatinine)	0.23 (0.16, 0.34)	<0.0001	20.45	0.26 (0.17, 0.41)	<0.0001	20.55	0.13 (0.05, 0.32)	<0.0001	26.49
	Model 2	Log(Urinary Creatinine)	0.22 (0.15, 0.33)	<0.0001	22.27	0.26 (0.16, 0.40)	<0.0001	21.86	0.10 (0.03, 0.27)	<0.0001	33.01
		eGFR	1.02 (1.01, 1.03)	0.001		1.01 (1.00, 1.03)	0.01		1.04 (1.01, 1.07)	0.003	
**Linear models**											
**Outcome**	**Model** a	**Predictor**	**B (SE)**	**P**	**R^2^** **(%)**	**B (SE)**	**P**	**R^2^** **(%)**	**B (SE)**	**P**	**R^2^** **(%)**
**%uMMA**	Model 1	Log (Urinary Creatinine)	−1.22 (0.34)	0.0004	16.06	−0.83 (0.39)	0.03	15.72	−2.77 (0.73)	0.0002	21.76
	Model 2	Log(Urinary Creatinine)	−1.21 (0.34)	0.0004	16.18	−0.83 (0.39)	0.03	15.82	−2.75 (0.73)	0.0003	21.84
		eGFR	−0.01 (0.01)	0.43		−0.01 (0.01)	0.51		−0.01 (0.02)	0.74	
**%uDMA**	Model 1	Log (Urinary Creatinine)	5.72 (0.57)	<0.0001	20.52	5.34 (0.67)	<0.0001	17.87	7.05 (1.15)	<0.0001	30.73
	Model 2	Log(Urinary Creatinine)	5.74 (0.58)	<0.0001	20.62	5.34 (0.67)	<0.0001	17.87	7.27 (1.13)	<0.0001	34.12
		eGFR	−0.01 (0.02)	0.44		−0.00 (0.02)	0.99		−0.08 (0.04)	0.02	

a We examined confounding or mediation of associations between uCrn and %As metabolites by using nested models, with and without control for eGFR; Model 1 parameters are log(age), sex, current smoking, log(total uAs), log(uCrn), and recruitment year (in total sample only); Model 2 parameters are log(age), sex, current smoking, log(total uAs), log(uCrn), eGFR, and recruitment year (in total sample only).

b Generalized R^2^.

c Probability modeled is %uInAs >12.2 (total sample: %uInAs ≤12.2 N = 168, %uInAs >12.2 N = 310; 2001 sample: %uInAs ≤12.2 N = 123, %uInAs >12.2 N = 245; 2003 sample: %uInAs ≤12.2 N = 45, %uInAs >12.2 N = 65).

We used multinomial logistic regression models to examine the pattern of association of uCrn and eGFR with the %uAs metabolites. A multinomial logistic model in the 2001 sample categorizing %uInAs at tertiles revealed a threshold effect of eGFR on %uInAs, in which eGFR was associated with increased odds of having %uInAs above the first tertile, while this positive association could not be observed with linear regression. Since the effect for the upper tertile vs. lower tertile was similar to that for the middle tertile vs. lower tertile, we dichotomized %uInAs at the lower tertile (at 12.2%, derived from the 2001 sample) to create a binary outcome for logistic regression. Linear regression models were applied to the continuous outcomes of %uMMA and %uDMA. Adding eGFR into the models did not appreciably change the estimated parameter of uCrn in any of the models. Results were similar when including water As as a covariate in these models in place of total uAs (data not shown).

As mentioned above, eGFR was positively associated with %uInAs in partial Spearman correlations within both samples (**Table 4**); upon covariate adjustment in regression models, these positive associations remained significant (**Table 5**). An increase in eGFR was associated with increased odds of having %uInAs above 12.2% (OR = 1.02, 95% CI (1.01, 1.03)). In the 2003 sample, the positive association between eGFR and log(%uInAs) was linear and significant when modeled with linear regression; a one unit increase in eGFR was associated with a 0.7% increase in %uInAs (b = 0.007, p<0.0001; data not shown). eGFR was not associated with %uMMA (b = −0.01, p = 0.43), and this was consistent in both the 2001 and 2003 samples. Finally, in the 2003 sample, a one unit increase in eGFR was associated with a mean decrease in %uDMA of 0.08 (b = −0.08, p = 0.02) in the covariate-adjusted model, however this association was not observed in the total sample or the 2001 sample.

## Discussion

For our first objective, we aimed to explore the potential renal toxicity of As. While water As was not associated with eGFR, we observed a marginally significant negative association between total uAs and eGFR in the total sample; this association remained marginally significant in the 2003 sample, while the association was negative but not significant in the 2001 sample. Additionally, the concentration of uMMA was a significant negative predictor of eGFR in the total sample, while uDMA was a negative predictor of eGFR in the 2003 sample only. The negative association between total uAs and eGFR in the 2003 sample might be attributed to the concentration of uMMA and uDMA, while in the 2001 sample the negative association may have been attributed to the concentration of uMMA. The 2003 sample had significantly higher total uAs concentrations, and uAs metabolite concentrations, relative to the 2001 sample, which may explain the stronger associations in the 2003 sample. Since the majority of total uAs consists of DMA, it is not surprising that the negative association between total uAs and eGFR in the 2003 sample would be attributed to uDMA. On the other hand, the negative association between uMMA concentration and eGFR may be due to the particularly high toxicity of MMA, as has been demonstrated in *in vitro* [58] and human studies [59].

Our finding of a negative association between total uAs and eGFR is consistent with other studies showing a negative association between total uAs and eGFR [9], [14], [15] or a positive association between total uAs and serum cystatin C [60]. A prospective study among children in Bangladesh observed a marginal negative association between total uAs at infancy and eGFR at 4.5 years old [61]. Total uAs has also been associated with increased urinary albumin [62], [63] and protein [64], and markers of renal tubular damage including increased urinary β_2_ microglobulin [15], [62], [65] and urinary *N*-acetyl-β-_D_-glucosaminidase (NAG) [62], [66]. Arsenic exposure has been associated with elevated mortality rates from kidney diseases in several populations [10]–[13]. Given the negative direction of association between uAs and eGFR, it is unlikely that this finding can be explained by the influence of GFR on the excretion of As in urine. Our finding is suggestive of the renal toxicity of As exposure, as measured by urinary As.

Although few studies have evaluated the prevalence of kidney diseases in Bangladesh, the CKD prevalence in our population in Araihazar was high in comparison with another Bangladeshi population located in Mirpur, Dhaka [67]. Huda et al. (2012) estimated GFR from serum creatinine using the Modification of Diet in Renal Disease (MDRD) equation in N = 1,000 adults in Mirpur, and found an average CKD prevalence of 13.1%. There may be several explanations for the higher CKD prevalence in our study. The first is that we used an ELISA method to measure cystatin C, which could potentially result in some misclassification given certain limitations in the precision of the technique. However, we do not believe that this alone could account for the large difference. The second explanation is that GFR estimating equations have not been extensively validated in South Asian populations, and may over- or under- estimate the true prevalence of CKD. A study in Pakistan found that the MDRD equation overestimated GFR as compared with a creatinine clearance test [68], which may explain why the Mirpur study had higher eGFR on average. The CKD-EPI equation that we have utilized has not yet been validated in a South Asian population. The reason for the difference in eGFR between the 2001 and 2003 samples remains unclear.

We also compared the average cystatin C in the 2001 and 2003 samples with age and sex stratified cystatin C averages from the third United States National Health and Nutrition Examination Survey (NHANES) [69]. Across all age and sex categories outlined in the NHANES study for which we had sufficient sample size (N>5), average cystatin C in both the 2001 and 2003 samples was higher than in NHANES, with the exception of 20–39 year old women in the 2003 sample who had similar cystatin C to the respective NHANES women. Consistent with the NHANES data, in our population cystatin C was higher in males than in females in every age category.

For our second objective, we aimed to determine whether GFR may explain the strong and consistent positive associations between uCrn and uAs methylation in this and previous studies [27]–[35]. We considered that GFR may explain these associations in one of two ways: one, that GFR may differentially influence the excretion of the As metabolites, and also influence creatinine excretion (i.e. confound the association), or two, that the As metabolites may differentially influence GFR, which would then influence creatinine excretion (i.e. mediate the association). Given our cross-sectional analysis, we are not able to distinguish between a confounding or meditational effect. However, adjustment for eGFR did not attenuate these associations, indicating that glomerular filtration does not explain the strong associations between uCrn and the %uAs metabolites (i.e. it is neither a confounder nor a mediator). eGFR was not associated with uCrn; this is not surprising, given that uCrn concentration is not a sensitive marker of renal function. Serum creatinine itself is not sensitive to moderate reductions in GFR [70]. Although creatinine is freely filtered at the glomerulus at a constant rate, it is secreted by the proximal tubular cells into the urinary space at a non-constant rate. When GFR decreases, tubular secretion of creatinine increases to offset the rise in serum creatinine [70]. This non-constant tubular secretion of creatinine, in addition to the strong dependence of uCrn on hydration status, is likely the reason we observed no association between eGFR and uCrn.

The strong associations between uCrn and %uAs metabolites may instead be due to the shared metabolic pathway of methylated As metabolites and creatine, as creatine biosynthesis is produced via the methylation of guanadinoacetate [71], a process that consumes a considerable amount of SAM [38]. Alternatively, it is possible that uCrn and the uAs metabolites are related due to similar renal handling in the proximal tubule. In humans, uCrn secretion is mediated by the organic cation transporter OCT2 at the basolateral membrane, and by P-glycoprotein 1 (P-gp, also known as multidrug resistance protein 1 (MDR1) or ATP-binding cassette sub-family B member 1 (ABCB1)) at the apical membrane [72]–[74], however little is known regarding the transporters mediating As secretion, and whether these transporters may have differential affinity for the different As species. There is evidence that cellular efflux of arsenite may be mediated by P-gp. Rat liver epithelial cells with acquired As tolerance have reduced As accumulation and increased expression of P-gp, while P-gp inhibition abolishes this tolerance [75]. Additionally, P-gp knockout mice are more sensitive to As-induced lethality and tissue damage, and accumulate more As in tissues (including kidney) following arsenite administration, than wild type mice [76]. The mutual utilization of P-gp by As and creatinine provides a potential link between As and creatinine secretion. Finally, creatinine may be associated with the urinary As metabolite profile circumstantially: experiments in dogs have revealed that arsenate reabsorption in the proximal tubule is sensitive to urine flow, with increased urine flow depressing arsenate reabsorption [44]. Since increased urine flow would result in enhanced arsenate excretion, and decreased uCrn (more diluted urine), this could result in a negative association between %uInAs and uCrn. Further research in this area is warranted to determine which transporters are responsible for As reabsorption and secretion within the proximal tubule, whether As may interfere with creatinine secretion or vice versa, and whether urine flow may be an important modifier of As reabsorption in humans. A limitation of this study is that we have not measured biomarkers of proximal tubular damage, so we are unable to examine the effects of As exposure and As methylation capacity on the proximal tubule, and the influence this may have on uCrn.

Interestingly, in both the 2001 and 2003 samples we found that eGFR was positively associated with %uInAs, independently of uCrn. Additionally, we observed that eGFR was negatively associated with %uDMA in the 2003 sample only. Given that this was a cross-sectional study, there are two potential explanations for these findings aside from unmeasured confounding: (a) GFR differentially influences the renal excretion of the As metabolites, and (b) the As metabolites are differentially detrimental to kidney function. We discuss both possibilities below.

### Possibility (a): GFR differentially influences the renal excretion of As metabolites

From the experiments of Ginsburg (1963) on the renal handling of arsenate in dogs, we know that arsenate is reabsorbed in the proximal tubule, and that increased urine flow depresses arsenate reabsorption, i.e. enhances its excretion [44]. In adult cancer patients with varying degrees of renal impairment undergoing arsenic trioxide treatment, mild to severe renal impairment decreased the renal clearance of InAs, and reduced both total As excretion and the percentage of the As dose excreted as arsenite [77]. These data indicate that improved GFR may result in improved excretion of InAs, which could be the reason that we observe a positive association between %uInAs and eGFR. This reason may also explain why we observe a negative association between %uDMA and eGFR in the 2003 sample, since the sum of %uInAs, %uMMA, and %uDMA within each individual is equal to 100%. Unfortunately, little is known regarding the renal handling and excretion mechanisms of MMA and DMA, so it is unclear whether the association between %uDMA with eGFR could be attributed to this alternative explanation. In the adult cancer patients from the above-mentioned study, mild to severe renal impairment resulted in increased serum concentrations of MMA and DMA, suggesting reduced renal clearance of these metabolites, although this data was not explicitly provided [77].

### Possibility (b): the As metabolites are differentially detrimental to kidney function

It is unlikely that high InAs exposure would result in improved GFR. InAs exposure results in mitochondrial toxicity in rat and human kidney proximal tubule cells [78], [79]. Mice exposed to arsenite had increased urinary N-acetyl-β-D-glucosaminidase (NAG; a marker of tubular damage) and tubular atrophy in the renal medulla [80]. Exposure of dogs to arsenate resulted in degeneration of the renal tubular epithelium at the lowest dose used (0.73 mg/kg), while reductions in creatinine clearance were only observed at higher doses [81]. An important caveat of these *in vivo* experiments is that the doses used are very high and much shorter in duration relative to the As intake from drinking water by humans in Bangladesh and other parts of the world. Therefore it is yet unclear what effect prolonged low dose InAs exposure would have on indices renal function.

While we do not propose that the positive association observed in our study between %uInAs and eGFR is indicative of a beneficial effect, it may be that a certain As methylation profile is detrimental to renal function. Since the sum of %uInAs, %uMMA, and %uDMA within each individual is equal to 100%, if one percent metabolite increases, another decreases. We therefore cannot know what metabolite(s) may be driving the associations that we observe with eGFR. In human studies, increased %uMMA has been associated with increased odds for a variety of diseases, including atherosclerosis, bladder cancer, lung cancer, skin cancer, and skin lesions [59], [82]. We did not observe that %uMMA was associated with eGFR in this study, although the concentration of MMA in urine was associated with decreased eGFR. In the 2001 sample, a methylation pattern of decreased %uInAs is associated with reduced eGFR, and in the 2003 sample, a methylation pattern of decreased %uInAs and increased %uDMA is associated with reduced eGFR. Very few studies have examined the associations of As metabolites with renal function, and therefore little is known regarding the expected direction of these associations. One study in Taiwan found that urinary %MMA was associated with lower eGFR, while urinary %DMA was associated with greater eGFR, however these associations were fully attenuated in covariate-adjusted models [14]. A cross-sectional study in American Indians in the U.S. found the prevalence of albuminuria (indicating glomerular and/or tubular damage) to be significantly *reduced* in the 2^nd^–4^th^ quartiles of %uMMA, as compared with the lowest quartile, in an adjusted model [63].

While methylation of InAs to DMA is considered a detoxification mechanism [32], in that it is crucial for As elimination from the body [83], As methylation results in intermediate metabolites, such as MMA^III^ and possibly DMA^III^, which are more toxic than InAs^III^ and their pentavalent counterparts, MMA^V^ and DMA^V^ [58]. DMA has a short circulating half-life and is the most prevalent As metabolite found in urine [84], so it is possible that the kidney tubules are exposed to DMA to a greater extent than other tissues, and that this higher dosimetry may be detrimental to kidney function. Since we only see an association between eGFR and %uDMA in the 2003 sample, this finding requires confirmation in future studies. Differences in As exposure, nutrition, and methylation capacity between the 2001 and 2003 samples may have accounted for the discrepancy.

Strengths of this study include the measurement of a sensitive biomarker of GFR, cystatin C, the speciation of As metabolites, and the large sample size. An important limitation of this study was that we did not measure markers of tubular damage, and were therefore unable to determine the effects of As exposure and/or methylation capacity on the renal tubules. The results from this study indicate areas for future research. Although our results suggest that the associations between %uAs metabolites and uCrn are not explained by glomerular filtration, it is also possible that the As metabolites and creatinine may share a mechanism of renal secretion that has not yet been explored. Additionally, due to the cross-sectional nature of this study, we cannot discern whether renal function may differentially influence the excretion of As metabolites, rather than the As methylation profile influencing renal function. Our results suggest that total As exposure may be detrimental to renal function; however, discrepant findings between our two study samples indicate a need for confirmation of this in future studies. Prospective studies examining the association between As exposure, As methylation capacity, and CKD incidence are warranted.
